# Rudolf Virchow and the discovery of the Müller cell

**DOI:** 10.1007/s00417-024-06615-6

**Published:** 2024-09-05

**Authors:** Helmut Kettenmann

**Affiliations:** 1 Max-Delbrück Center for Molecular Medicine, Berlin, Germany; 2 Shenzhen University of Advanced Technology, Shenzhen, China

**Fig. 1:**
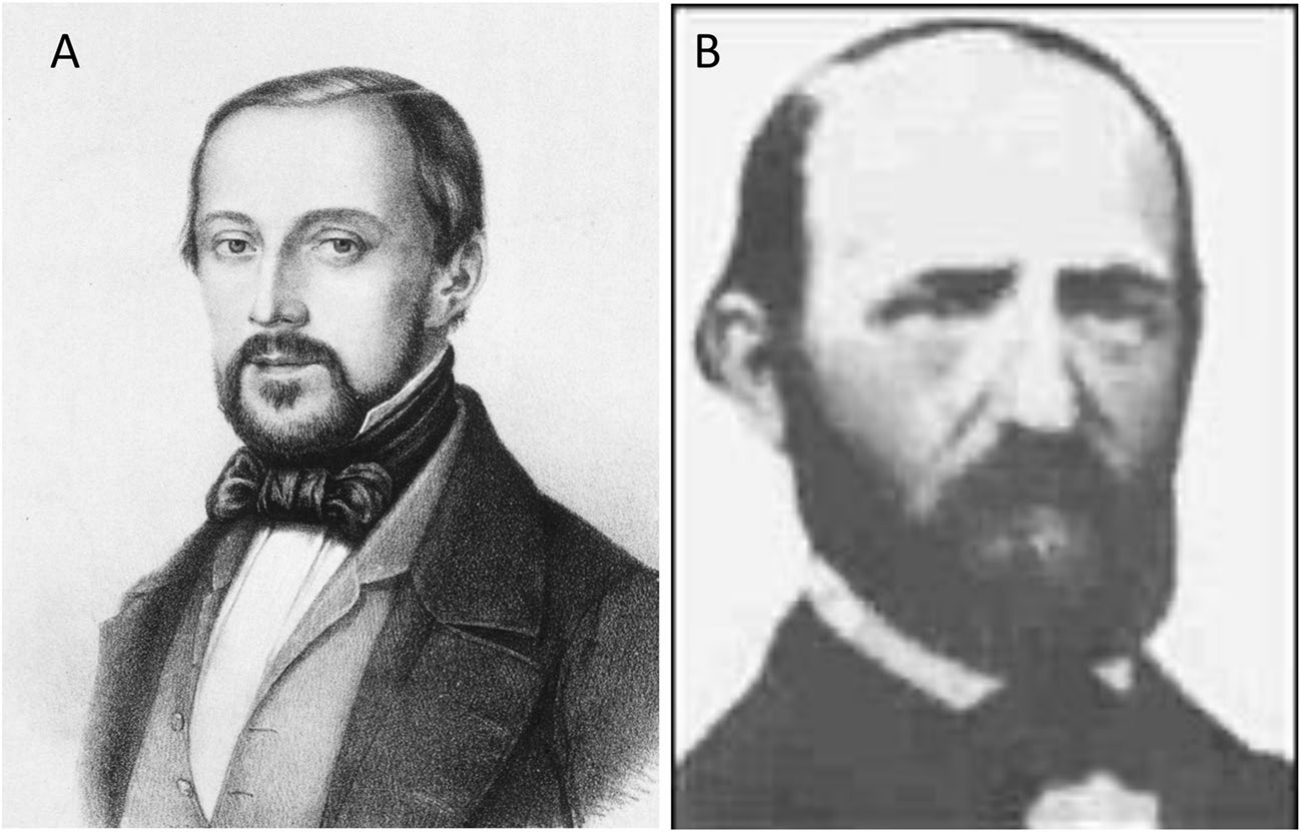
Portrait Virchow and Müller. **A** Portrait of Rudolf Virchow (from Wikipedia, public domain). **B** Portrait of Heinrich Müller (From WürzburgWiki.de)

Albrecht von Graefe (1828–1870) studied medicine in Berlin and one of his teachers was Rudolf Virchow (1821–1902; Fig. 1A) who later became his colleague at the Charité. Graefe was appointed as Director of the Department of Ophthalmology in 1868. At that time Virchow has been Chair of the Department of Pathology since 1856.

Both founded scientific journals. In 1847—at the age of 26—Virchow established the *Archiv für pathologische Anatomie und Physiologie und für klinische Medicin*, published by Georg Reimer. He remained Editor-in-Chief until his death in 1902. Afterwards, the journal was renamed to *Virchows Archiv für pathologische Anatomie und Physiologie und für klinische Medizin*. It still exists as *Virchow Archiv,* published by SpringerNature.

Graefe founded his journal in 1854—also at the age of 26—and both journals belong to the oldest, still existing medical journals.

Virchow’s fame is linked to his seminal book *Cellularpathologie* published in its first edition in 1858. After the cell theory became established in Berlin in 1838/1839 by Matthias Schleiden (1804–1881) for plants and Theodor Schwann (1810–1882) for animals postulating that all tissues are composed of cells as a basic unit, it was Rudolf Virchow who established the concept that one can diagnose diseases by analyzing the cells in the tissue of a patient which is the basis of today´s histological diagnosis. The *Cellularpathologie* is the blueprint of that concept. Another important concept published in that book is the vision of glial cells. Virchow recognized there are two major cell types in the brain, neurons and glial cells. Neurons were first depicted by Christian Ehrenberg (1795–1876) in 1836, another influential scientist at the Berlin University. The merit of Virchow was that he coined the name ´glia´ from the Greek meaning putty or glue. The old concept was that this should be the filling material of the brain. Virchow's published figures illustrating his "new" tissue (and the cells it contained) in the *Cellularpathologie* are generally considered as the first images of glial cells (Fig. 3A, B). These illustrations show what are presumed to be "connective­ tissue-corpuscles and nuclei" beneath ependymal cells in the floor of the fourth ventricle.

While most historians award Virchow exclusive credit for first recognizing glial cells, another pathologist published a picture of a glial cell even before Virchow, and his picture was vastly superior in clarity (Fig. 3C, D). It was obtained from the eye, more specifically from the retina. Heinrich Müller (1820–1864; Fig. 1B) already noted in 1851 that the retina contains radial fibers [3], and in 1852 his colleague, Albrecht von Kölliker (1817–1905) termed these structures Müller fibers [2]. Köllikers writes: ´*It is difficult to finally reveal the features of these fibers in the granule cell layer. It is easy to follow their course in the layer of ganglion cells and the nerve layer until the inner surface of the last layer, but nobody studied vertical section of the retina and it is difficult to explain why everybody overlooked this radial fiber system or the Müller fibers as I would name them after his discoverer´.* In his detailed article, Müller compares the retina in the fish (perch), amphibia (frog), bird (pigeon), and human. In his summary he states: ´*The presence of radial fibers is a common feature. In all cases, they extend from the inner surface of the retina to the inner granular layer where they show a swelling containing a nucleus; from there they continue to the outer layers.´* Müller had clearly recognized that these fibers are part of a cell with the soma in the inner granular layer containing a nucleus,. This is convincingly supported by his figures which are, in fact, the first documentation of a glial cell (Fig. 3D). Still, one must credit Virchow for introducing the theory of a neuroglial tissue, even if "the theory led and the facts followed" [1]. It is this theory or idea, more than anything else, that secures for Virchow his special place in the history of this field.

It is an irony of history that Virchow was the reason why Heinrich Müller changed his scientific field from cancer research to the histology of the retina which led to the discovery of the Müller cell. Heinrich Müller, one year older than Virchow, was a senior assistant in the department of pathology at Würzburg University with a research focus on cancer. He had studied medicine in München, Freiburg and Würzburg, spending also some time in Wien and Heidelberg. There he was introduced to microscopy by Jakob Henle (known from the Henle loop in the kidney) who worked in the scientific environment with Schwann and Schleiden, but had moved from Berlin to Heidelberg. In Würzburg Müller joined the Department of Pathology and did his second degree (Habilitation) on the topic ´On the nature of neoplasm in particular tumor and blood coagulation´. After the sudden death of the Chair of the Department of Pathology, Bernard Mohr, due to tuberculosis in winter of 1848, he took over the Chair of Pathology as deputy. Most of his colleagues in the faculty favored him as the next chair holder.

**Fig. 2:**
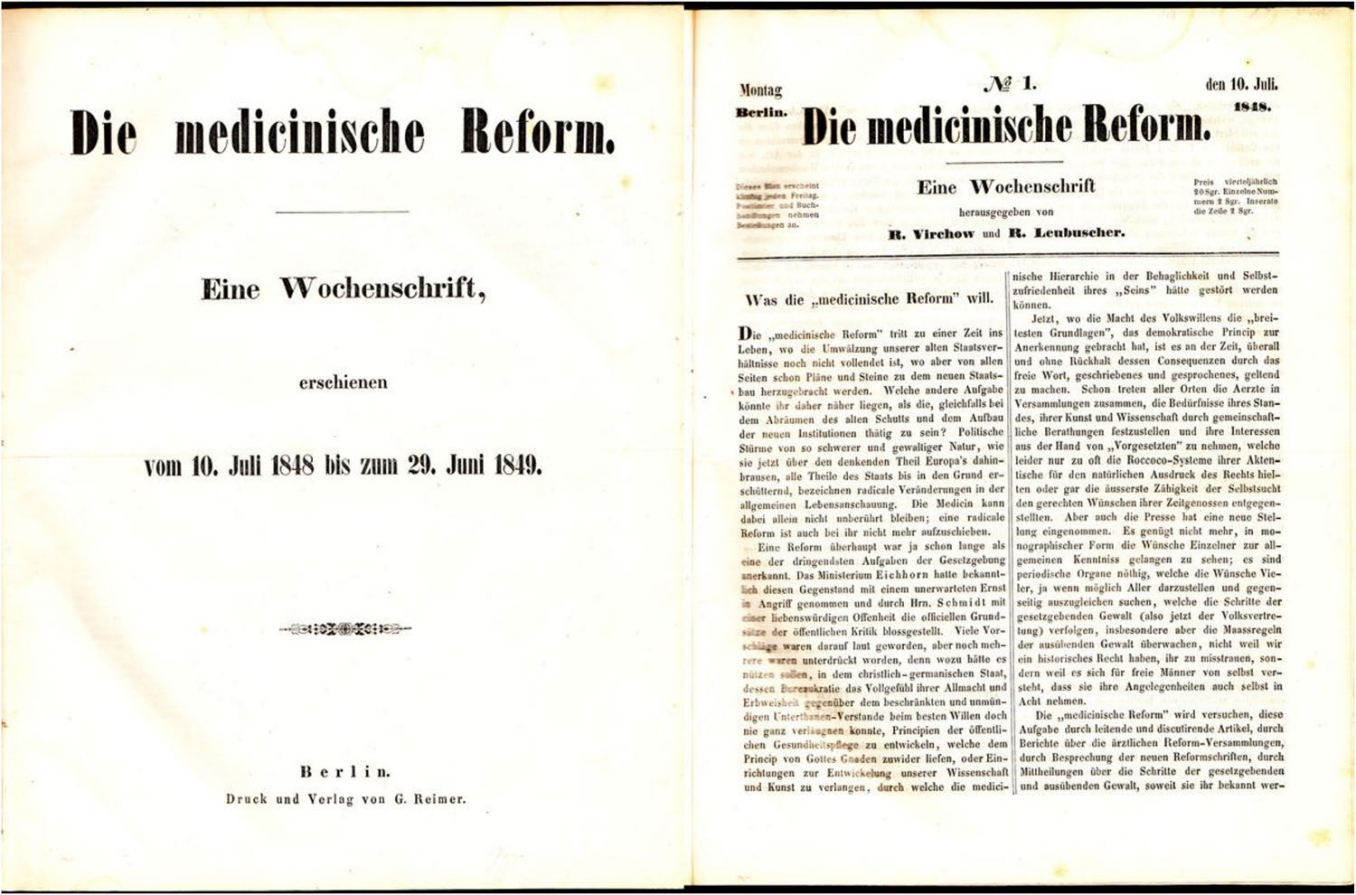
Frontpages of ´Die medicinische Reform´

At the same time that this nomination process was moving forward, Virchow was involved in the 1848–1849 revolution. He participated in erecting barricades in the famous March days of 1848. He also intended to extend the revolution to medicine. Together with Rudolf Leubuscher, he founded a new weekly magazine, *Die medicinische Reform* (the medical reform), which was launched on June 10, 1848 (Fig. 2). The articles in this magazine, several written by Virchow, formulated the concept that medicine should be available to all citizens and that it should be based on a scientific ground. With the end of the revolution, Virchow´s situation in Berlin became difficult. He lost his job as prosector at the Charité and his apartment. *Die medicinische Reform* drew to a close. In the last issue published in June 29,1849, it was reported under the personal notes section that Drs. Waldeck and Weiss were imprisoned for three month and that the members of the medical central committee from Dresden, Prof. Richter and Dr. Seidenschnur were in now prison.

Therefore, he had to find a job elsewhere. He was, however, already well-known and several universities including the Eidgenössische Technische Hochschule (ETH) in Zurich offered him a chair.

The faculty in Würzburg realized their chance and offered Virchow—and not Müller—the Chair of Pathology. Virchow accepted the position. Seven years later he returned to Berlin to become professor of pathology at the Charité. Müller was so depressed by this unfortunate turn of events that he had to recover for half a year in a sanatorium in Boppard and in Southern France. After his return to Würzburg, he decided to switch to comparative histology and joined Albrecht von Kölliker's Department of Anatomy and Histology. There he began his thorough analysis of the anatomy and physiology of the eye with a strong focus on histology. His studies on the cellular composition of the retina were ground-breaking. All these studies were carried out on unstained tissue since the first dye, carmine red had just been introduced. The cells were isolated by very carefully teasing the tissue apart with fine needles and thereby trying to isolate single cells or ensembles of a few cells. Of course, this was much easier in the retina than in the whole brain and explains why the first modern pictures of glial cells came from the retina. Müller’s thorough analysis of the retina yielded several discoveries such as that some birds had two foveae. Inspired by a visit to Albrecht von Graefe in Berlin in 1854, he extended his research also to the pathology of the eye. In his paper published in 1856 on Morbus Brighti, he writes at the beginning: ´*Due to the courtesy of von Gräfe in Berlin, I had the opportunity to study the chorioidea of a 12-year old child which had died due to Brighti´s disease*´. Both, Graefe and Müller, died very early at the peak of their careers, Graefe at 42, Müller at 44 years.

**Fig. 3:**
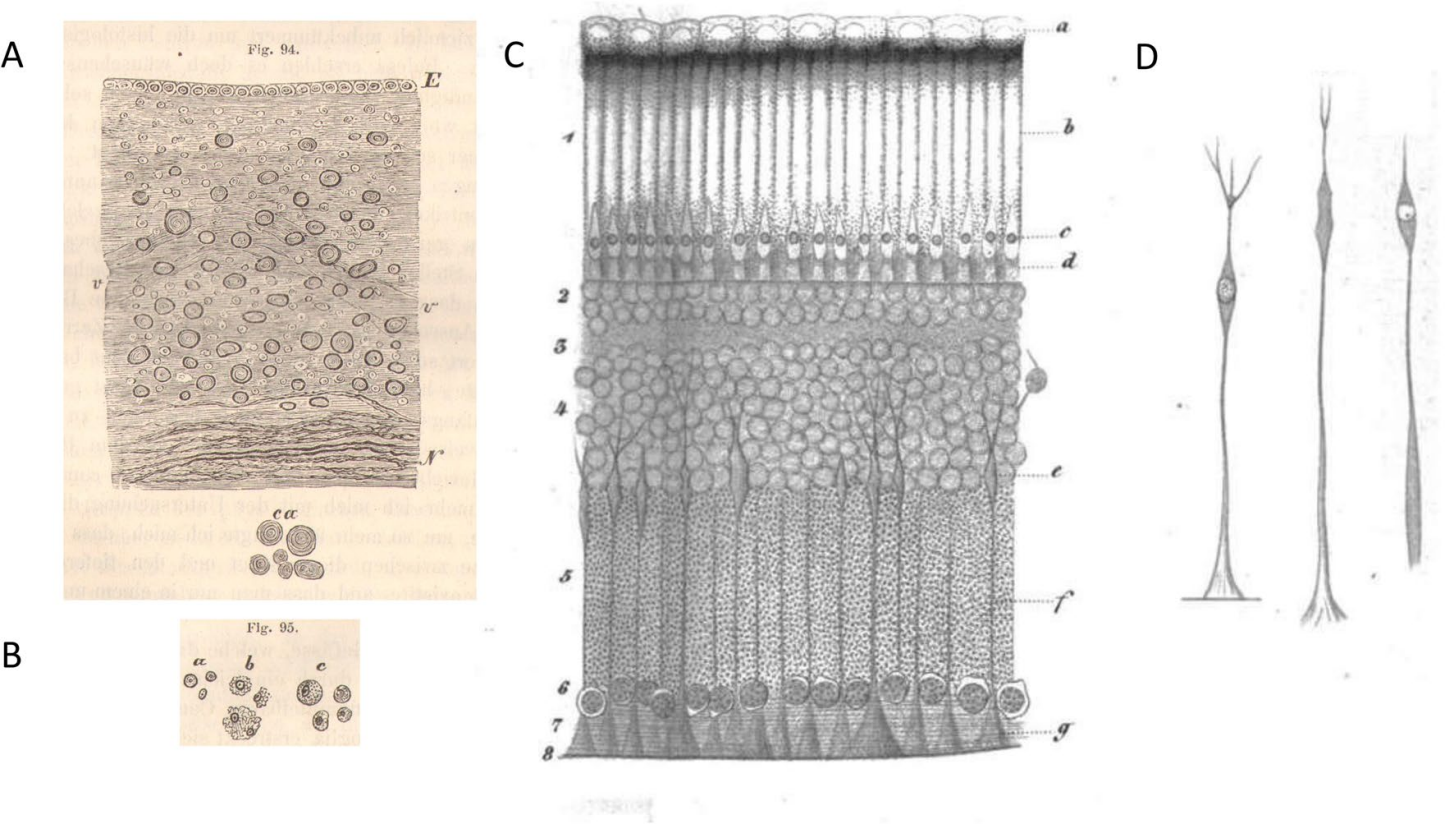
Image of neuroglia and Müller cells. **A** Virchow’s illustration of neuroglia. A. Ependyma and neuroglia in the floor of the fourth ventricle. Between the ependyma and the nerve fibers is “the free portion of the neuroglia with numerous connective tissue, corpuscles and nuclei.” Numerous corpora amylacea are also visible, shown enlarged in below the main illustration (ca). E: ependymal epithelium; N: nerve fibers; v-w: blood vessel (From Virchow [6]). **B** Elements of neuroglia from white matter of the human cerebral hemispheres. A. free nuclei with nucleoli; b, nuclei with partially destroyed cell bodies; c: complete cells (From Virchow [6]). **C** Vertical section of the retina of the frog. a) pigment cells with their nuclei; b) cones; c) rods; d) border layer between rod layer and granule layer; e) swelling of the radial fibers f) whose conic ends g) end at the limitans. 1 layer of rods; 2 outer granular layer; 3 intermediate granular layer; 4 inner granular layer; 5 granulous layer; 6 nerve cell layer; 7 fibers of the optic nerve; 8 border layer (From Müller [4]). **D** Isolated radial fibers from frog (From Müller [4])

Heinrich Müller, however, will be unforgotten due to his discovery and definition of the principal glial cell of the retina, the Müller cell (Fig. 3).
