# Correction: Rudolf Virchow and the discovery of the Müller cell

**DOI:** 10.1007/s00417-024-06689-2

**Published:** 2024-11-12

**Authors:** Helmut Kettenmann

**Affiliations:** 1 Max-Delbrück Center for Molecular Medicine, Berlin, Germany; 2 Shenzhen University of Advanced Technology, Shenzhen, China

**Correction: Graefe's Archive for Clinical and Experimental Ophthalmology**

10.1007/s00417-024-06615-6

In the published original article, the order of the affiliation is incorrect.

The correct order would be:

^1^ Max-Delbrück Center for Molecular Medicine, Berlin, Germany

^2^ Shenzhen University of Advanced Technology, Shenzhen, China

The original article has been corrected.

